# The Clinical Value of Procalcitonin in the Neutropenic Period After Allogeneic Hematopoietic Stem Cell Transplantation

**DOI:** 10.3389/fimmu.2022.843067

**Published:** 2022-04-25

**Authors:** Meng Shan, Danya Shen, Tiemei Song, Wenyan Xu, Huiying Qiu, Suning Chen, Yue Han, Xiaowen Tang, Miao Miao, Aining Sun, Depei Wu, Yang Xu

**Affiliations:** ^1^ Jiangsu Institute of Hematology, The First Affiliated Hospital of Soochow University, Suzhou, China; ^2^ National Clinical Research Center for Hematologic Diseases, The First Affiliated Hospital of Soochow University, Suzhou, China; ^3^ Institute of Blood and Marrow Transplantation, Soochow University, Suzhou, China; ^4^ Collaborative Innovation Center of Hematology, Soochow University, Suzhou, China

**Keywords:** procalcitonin, allogeneic hematopoietic stem cell transplantation, infectious disease, prognosis, nomogram

## Abstract

The diagnostic value of procalcitonin and the prognostic role of PCT clearance remain unclear in neutropenic period after allogeneic hematopoietic stem cell transplantation introduction. This study evaluated 219 febrile neutropenic patients (116, retrospectively; 103, prospectively) who underwent allo-HSCT from April 2014 to March 2016. The area under the receiver operator characteristic curve (AUC) of PCT for detecting documented infection (DI) was 0.637, and that of bloodstream infection (BSI) was 0.811. In multivariate analysis, the inability to decrease PCT by more than 80% within 5–7 days after the onset of fever independently predicted poor 100-day survival following allo-HSCT (P = 0.036). Furthermore, the prognostic nomogram combining PCTc and clinical parameters showed a stable predictive performance, supported by the C-index of 0.808 and AUC of 0.813 in the primary cohort, and C-index of 0.691 and AUC of 0.697 in the validation cohort. This study demonstrated the diagnostic role of PCT in documented and bloodstream infection during the neutropenic period after allo-HSCT. PCTc might serve as a predictive indicator of post-HSCT 100-day mortality. A nomogram based on PCTc and several clinical factors effectively predicted the 100-day survival of febrile patients and may help physicians identify high-risk patients in the post-HSCT neutropenic period.

## Introduction

Infectious disease remains a major complication of allo-HSCT, especially in the post-HSCT neutropenic period. These profoundly immunocompromised patients in the neutropenic period are susceptible to fatal infection. Early diagnosis and initiation of antibiotic treatment is the keystone of infectious disease. Owing to similar clinical symptoms, such as fever, distinguishing infectious diseases from non-infectious transplant-related complications is challenging.

Previous studies have revealed the usefulness of PCT for diagnosis of bacterial infections with high specificity in neutropenic patients (1–4). PCT-guided treatment achieved a significant reduction in antibiotic exposure in critically ill patients with a presumed bacterial infection, and improved the survival of patients (5–7). Additionally, PCTc is recognized as a prognostic marker in severe sepsis patients (8–10). Serial PCT elevation has been associated with a high risk of mortality. However, the diagnostic value of PCT in allo-HSCT remains controversial. Several studies have shown that PCT has little value in the diagnosis of infection, or is not superior to other infectious biomarkers, such as CRP (11, 12). One possible explanation might be the marked increase in PCT induced by the conditioning regimen (13, 14). Therefore, our study focused on febrile patients in the neutropenic period, avoiding interference from the conditioning regimen. Moreover, the relationship between PCTc and post-HSCT survival has not been well established in allo-HSCT. This study was designed to assess the diagnostic value of PCT for infection and to evaluate whether PCTc is associated with survival outcomes in neutropenic patients following allo-HSCT. Based on the results, we developed a nomogram incorporating PCTc into selected clinical parameters. This nomogram could be a useful prognostic tool for patients, physicians, and clinical investigators.

## Materials and Methods

### Study Design and Patients

**Figure 1:**
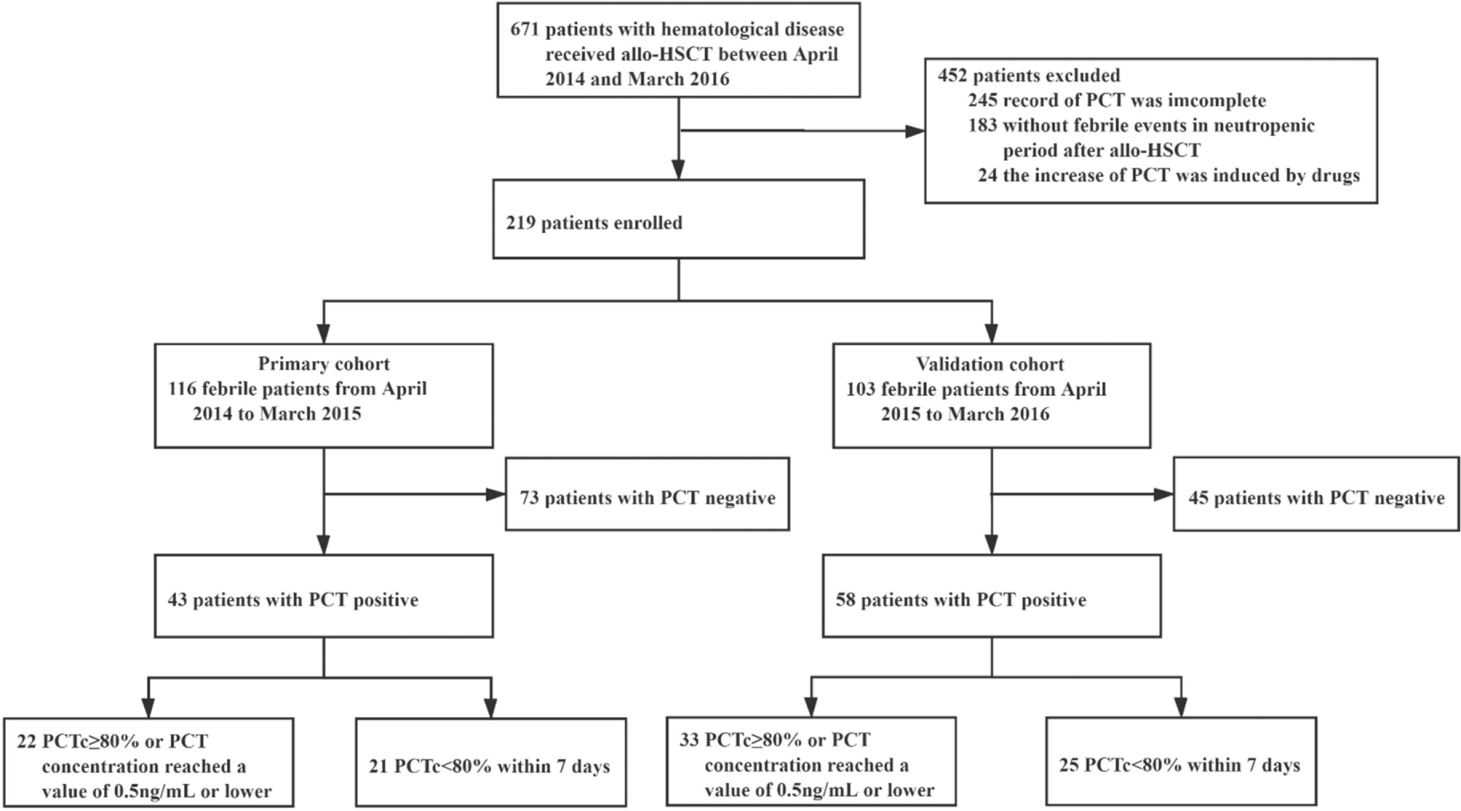
The study profile.

A retrospective study was conducted on a primary cohort of 116 patients with hematological disease undergoing allo-HSCT from April 2014 to March 2015 at the First Affiliated Hospital of Soochow University (**Figure 1**). The inclusion criteria included the following: 1) patients who underwent allo-HSCT; 2) patients who became febrile and had complete records of PCT values (measured weekly from admission to discharge and at the onset of fever) in the neutropenic period after allo-HSCT; and 3) patients with complete clinical data and follow-up information. The exclusion criteria were as follows: 1) for patients who were PCT positive, PCTc could not be evaluated 5–7 days after the first positive PCT test; and 2) an increase in PCT induced by drugs, such as antithymocyte globulin (ATG) (13, 14).

From April 2015 to March 2016, 103 patients were studied prospectively, using the same inclusion and exclusion criteria. These patients formed the validation cohort of this study.

### Conditioning Regimen for HSCT and Graft-Versus-Host Disease Prophylaxis

For nonmalignant hematological disease, nine patients received Bu/Cy + ATG as a conditioning regimen. Two patients received the FC + ATG regimen. For malignant disease, 64 patients received the modified Bu/Cy regimen. Thirty-two malignant patients received the Bu/Cy regimen. Nine malignant patients received the TBI/Cy regimen. ATG was included in the conditioning regimen of 84 malignant hematological patients.

Graft-versus-host disease (GVHD) prophylaxis regimens consisting of cyclosporine and short-range methotrexate were used for HLA-matched donors, while cyclosporine, methotrexate, and mycophenolate mofetil were used for HLA-mismatched donors.

The protocols of conditioning regimen and graft-versus-host disease prophylaxis are described in **Supplemental Material**.

### Infection Prophylaxis

Broad-spectrum penicillins (piperacillin-tazobactam) or cephalosporins (cefoperazone sodium and sulbactam sodium) were administered as infection prophylaxis for bacterial infection. When patients were febrile with severe clinical presentation or previous infection due to extended-spectrum β-lactamase producing or multi-drug resistance bacteria, the therapy was escalated to or combined with a broader-spectrum antibiotic, such as carbapenems and colistin. Vancomycin or linezolid was added to cover Methicillin-resistant *Staphylococcus aureus*. Once there is no microbiologically evidence or the pathogen was defined as its susceptibility profile or patient meliorated, the therapy was then de-escalated to narrower-spectrum antibiotics. The antibiotic treatments were all escalated at the onset of fever in PCT-positive patients during post-HSCT neutropenic period and de-escalated by clinicians according to clinical manifestation and laboratory examination of patients after antibiotic treatments.

Sulfa-methoxazole-trimethoprim was administered for *Pneumocystis jiroveci* infection from admission until neutrophils were above 0.5 × 10^9^/L. Fluconazole or voriconazole was given for fungal infection until the end of immunosuppressive drugs. Acyclovir as prophylaxis against herpes infection was administered from the start of conditioning until one year after allo-HSCT.

Granulocyte colony-stimulating factor at a dose of 5 mg/kg/d was given from day +7 until myeloid recovery (absolute neutrophil count ≥1.5 × 10^9^/L for 3 consecutive days).

### Sampling and Measurement

PCT was determined once a week from the start of conditioning to discharge or death. For each febrile event, we performed a blood culture from both a peripheral site and a central venous catheter or peripherally inserted central catheter and additional measurements of PCT at the onset of fever (onset PCT, O-PCT). Culture specimens from other sites of suspected infection were obtained according to the clinical indication (15). Reference values were <0.5 ng/ml for PCT.

PCTc was calculated using the following formula:


PCTc =[ O-PCT-PCT(reexamination)]/ O-PCT∗100%

### Definition and Outcome Assessment

A febrile event was defined as an axillary temperature of ≥38.3°C at the first onset of fever or an axillary temperature of ≥38.0°C, which lasted more than 1 h (except for the administration of drugs or blood-derived products). DI included microbiologically documented and presumed infections diagnosed by the clinician based on clinical, laboratory, and radiological examinations, and the clinical course and response to treatment were in accordance with an infectious etiology. BSI was confirmed by at least one positive blood culture, except for Corynebacterium species, Bacillus species, coagulase-negative staphylococci, and other skin commensals, which required at least two positive blood cultures (16). Cytomegalovirus (CMV) and Epstein–Barr virus (EBV) reactivations were diagnosed when DNA was detected.

Disease status was classified into low risk (leukemia or lymphoma in the first or second complete remission, myelodysplastic syndrome without excess blasts, and nonmalignant hematological disease in remission) and high risk (all other conditions).

The reconstitution of neutrophils and platelets after HSCT was defined as neutrophils not lower than 0.5 × 10^9^/L for more than 3 days and platelets not less than 20 × 10^9^/L for more than 7 days without transfusion (17). The assessment of GVHD was in accordance with the Modified Glucksberg criteria and Revised Seattle criteria (18). Major transplant-related complications (MTC) include III–IV aGVHD, veno-occlusive disease, thrombotic microangiopathy, and neurological complications.

### Statistical Analysis

Descriptive statistics were used to characterize the clinical information of patients and transplantation. Differences in continuously parameterized characteristics between two groups were assessed using a Mann–Whitney U test and among multiple groups using a Kruskal–Wallis test with subsequent pairwise comparisons *via* the Steel–Dwass method. X^2^ tests or Fisher’s exact tests were used to investigate differences in categorical features. OS and 100-day survival were estimated using the Kaplan–Meier method, and differences between groups were compared with a log-rank test. A Cox regression model was carried out to determine the factors associated with 100-day survival. Variables with a p-value of less than 0.2 at the univariate level were entered into the multivariable regression models. The receiver operating characteristic (ROC) analysis was used to assess the diagnostic value of the PCT level for DI or BSI after allo-HSCT.

The predictive nomogram was developed based on the risk features identified by Cox univariate analysis. The discrimination power of the nomogram was measured by the concordance index (C-index). The nomogram was applied to the validation set for external validation. Predicted survival was compared with the actual condition using calibration plots in both the primary and validation cohorts (19). ROC curves and AUC analyses were also used to evaluate the performance of the models in the primary and validation cohorts. A two-sided P-value of 0.05 was considered statistically significant for these tests. For statistical analyses, SPSS 22.0 (SPSS, Chicago, IL, USA), R 3.6.1 (R Foundation for Statistical Computing, Vienna, Austria) and MedCalc Statistical Software version 12.7.0.0 (MedCalc Software bvba, Ostend, Belgium) were used.

## Results

### Characteristics of Patients and Transplantation

In the primary cohort, of the 267 patients with hematological disease who received allo-HSCT between April 2014 and March 2015, 116 met the inclusion criteria and were enrolled in this study. There were 73 patients who were PCT negative and 43 patients who were PCT positive once or more times during the study period. Among the 43 patients who were PCT positive, the PCT concentration in 22 patients decreased by 80% or more of its initial positive value or reached a value of 0.5 ng/ml or lower within 5–7 days, and in 21 patients, it decreased by less than 80% beyond 7 days (**Figure 1**). The baseline patient and transplantation characteristics in the primary and validation cohorts are summarized in **Table 1**.

**Table 1:** Baseline patient and transplantation characteristics.

Characteristics	Primary Cohort (n = 116)	Validation Cohort (n = 103)
	N (%)/Median (Range)	N (%)/Median (Range)
Age at transplantation, years (range)	28 (4–57)	26 (3–61)
Gender, n (%)		
Male	80 (69.0)	62 (60.2)
Female	36 (31.0)	41 (39.8)
Disease status, n (%)		
Low risk	95 (81.9)	76 (73.8)
High risk	21 (18.1)	27 (26.2)
HCT-CI, n (%)		
0–2	109 (94.0)	97 (94.2)
3 or more	7 (6.0)	6 (5.8)
ATG, n (%)		
Yes	84 (72.4)	88 (85.4)
No	32 (27.6)	15 (14.6)
Donor type, n (%)		
HLA-identical siblings	35 (30.2)	19 (18.4)
Matched unrelated donor	17 (14.7)	9 (8.7)
Haploidentical-related donor	63 (54.3)	74 (71.8)
Cord blood	1 (0.8)	1 (1.0)
Stem-cell source, n (%)		
BM	15 (12.9)	16 (15.5)
PBSC	46 (39.7)	20 (19.4)
BM + PBSC	54 (46.6)	66 (64.1)
Cord blood	1 (0.8)	1 (1.0)
Infused MNC^+^, *10^8^ cells/kg (range)	9.41 (2.30–29.31)	10.57 (1.82–23.50)
Infused CD34^+^, *10^6^ cells/kg (range)	3.62 (1.23–12.17)	3.82 (1.45–9.34)

HCT-CI, hematopoietic cell transplantation-specific comorbidity index; ATG, antithymocyte globulin; BM, bone marrow; PBSC, peripheral blood stem cell; MNC, mononuclear cell.

### Diagnostic Value of PCT for DI and BSI

**Figure 2:**
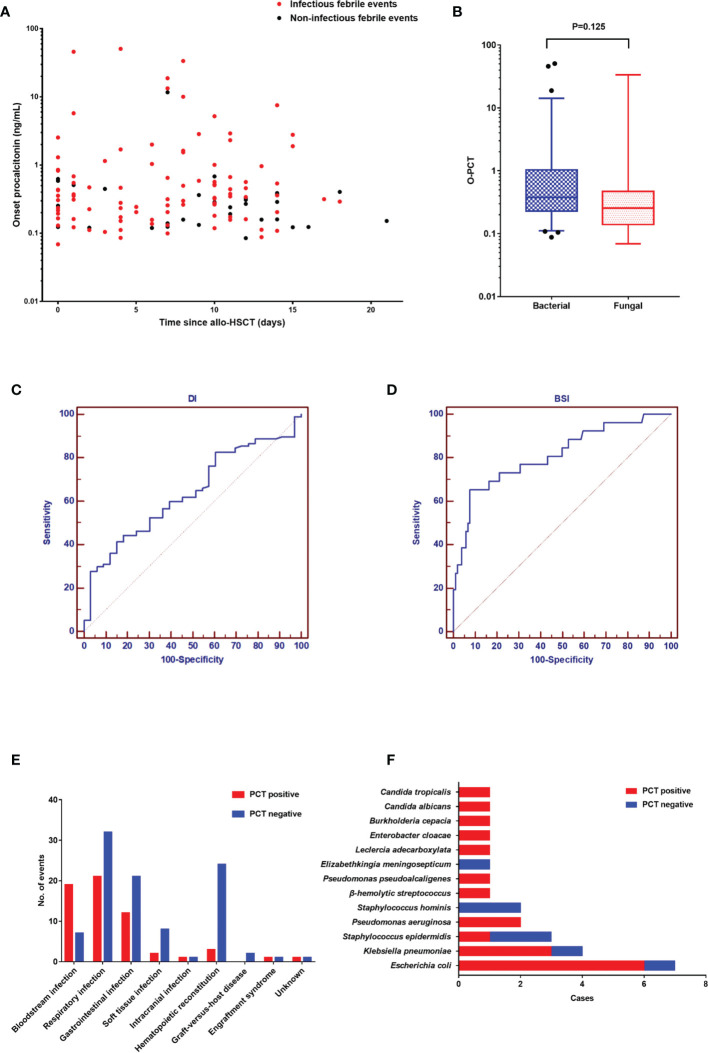
**(A).** The onset value of procalcitonin in 130 febrile events in the neutropenic period after allogeneic hematopoietic stem cell transplantation. **(B)** Comparison of the onset PCT value among the three groups: fungal infection, bacterial infection, and noninfectious events. **(C)** The diagnostic value of procalcitonin for documented infection. **(D)** The diagnostic value of procalcitonin for documented infection. **(E)** The diagnosis of 130 febrile events. **(F)** The spectrum of pathogens in 26 positive blood cultures.

In our study (**Figure 2A**), there were 130 febrile events in the neutropenic period after allo-HSCT in our study. Infectious events (97/130) constituted the most febrile events, with a median PCT of 0.35 ng/ml (range: 0.07–50.71), while noninfectious events had a median PCT of 0.27 ng/ml (0.09–11.70). The PCT level in infectious events was significantly higher than that in noninfectious events (P = 0.019). In **Figure 2B**, we compared the O-PCT among fungal infections, bacterial infections, and noninfectious events. Pairwise comparisons corrected for multiple testing showed significant differences only in PCT values between bacterial infection and noninfectious events (P = 0.013).

As shown in **Figures 2C, D**, the AUC of PCT was 0.637 (95% confidence interval [CI]: 0.549–0.720) for DI and 0.811 (95% CI: 0.733–0.875) for BSI. Diagnostic cutoff levels with the optimal sensitivity and specificity were 0.405 ng/ml for DI and 0.854 ng/ml for BSI. Referring to previous studies (1, 3, 4) and this study, the cutoff value of 0.5 has good sensitivity and specificity in the diagnosis of DI (sensitivity: 41.2%; specificity: 84.9%). Therefore, we defined a PCT value ≥0.5 ng/ml as positivity. The most common infection site was the respiratory tract (53/97), followed by the gastrointestinal tract (33/97) and soft tissue (10/97) (**Figure 2E**). The positive rate of BSI was higher than that of other sites. In 26 positive blood cultures, the common pathogens were *Escherichia coli* (7/26), *Klebsiella pneumoniae* (4/26), and *Staphylococcus epidermidis* (3/26) (**Figure 2F**). Moreover, although the difference did not reach statistical significance (P = 0.149), there were fewer gram-positive BSIs with PCT positivity (4/8) than gram-negative BSIs with PCT positivity (15/18).

### Engraftment and Transplant-Related Complications in the Primary Cohort

**Figure 3:**
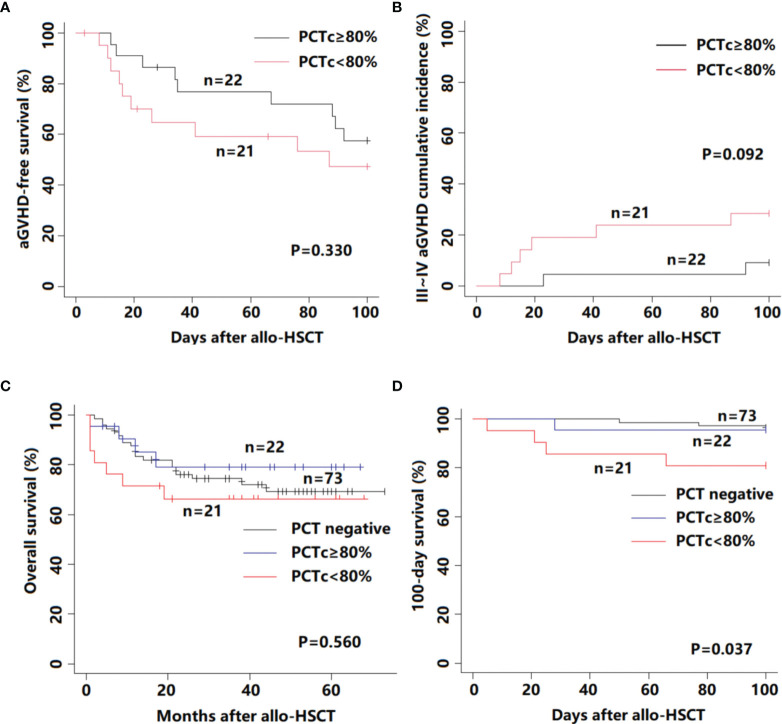
Survival outcome for 116 febrile patients in the neutropenic period after allo-HSCT according to PCTc. **(A)** aGVHD-free survival; **(B)** III–IV aGVHD cumulative incidence; **(C)** overall survival; and **(D)** 100-day survival.

A comparison of engraftment and transplant-related complications according to PCTc is listed in **Table S1**. Except for three patients who died without engraftment, the time to neutrophil and platelet reconstitution in the remaining patients among the three groups was not significantly different (neutrophil, P = 0.232; platelet, P = 0.205). The proportion of BSI in the PCT-positive group was significantly higher than that in the PCT-negative group (P <0.001), but the occurrence of transplant-related complications was not significantly different among the three groups (P >0.05). The aGVHD-free survival within 100 days post-HSCT between the PCTc ≥80% and PCTc <80% groups was not significantly different (P = 0.330, **Figure 3A**). In **Figure 3B**, although the difference did not reach statistical significance, the cumulative incidence of III–IV aGVHD in the PCTc <80% group within 100 days post-HSCT was higher than that in the PCTc ≥80% group (PCT ≥80% group: 9.1%; PCT <80%: 28.6%; P = 0.092).

### PCTc is Associated With the Occurrence of Post-HSCT 100-Day Mortality

In our study, among 116 patients with a median follow-up time of 35 months (range, 1 to 73 months), fourteen patients died of relapse of primary disease and 17 patients died of transplant-related mortality. There was no statistically significant difference between these three groups (P = 0.560, **Figure 3C**). We further analyzed the 100-day survival after allo-HSCT among the three groups. Death before day 100 post-HSCT occurred in 8 patients (3 patients in the PCT negative group, 1 in the PCTc ≥80% group, and 4 in the PCTc <80% group), and the primary causes were all transplant-related, namely, infection (n = 5), aGVHD (n = 2), and VOD (n = 1). The 100-day survival in the PCT <80% group was significantly lower than that in the other two groups (P = 0.037, **Figure 3D**).

Referring to previous studies (20–22), ten variables considered having potential prognostic value for 100-day survival were age, diagnosis (malignant/nonmalignant), disease status (low risk/high risk), donor type (HLA-matched/haploidentical), months between diagnosis and HSCT (<10/≥10), HCT-CI (0–2/3 or more), ATG (No/Yes), MTC (No/Yes), CMV(No/Yes), and PCTc (PCT negative/PCTc ≥80%, or PCT turned negative/PCTc <80%) (**Table 2**). In multivariate analysis, PCTc <80% was an independent factor with an adverse effect on 100-day survival (P = 0.036, HR = 5.697, 95% CI: 1.122–28.931).

**Table 2:** Univariable and multivariable analysis for 100-day survival of the primary cohort.

Variable	Univariable		Multivariable	
	Hazard ratio (95%CI)	p	Hazard ratio (95%CI)	p
Age	0.972 (0.920–1.028)	0.319		
Diagnosis (malignant/nonmalignant)	3.620 (0.730–17.944)	0.115	1.575 (0.122–20.258)	0.728
Disease status (Low risk/high risk)	2.841 (0.679–11.892)	0.153	2.958 (0.653–13.406)	0.160
Donor type (HLA-matched/haploidentical)	0.863 (0.216–3.452)	0.835		
Months between diagnosis and HSCT (<10/≥10)	3.870 (0.924–16.203)	0.064	4.783 (0.533–42.903)	0.162
HCT-CI (0–2/3 or more)	2.482 (0.305–20.178)	0.395		
ATG (No/Yes)	0.652 (0.156–2.727)	0.558		
MTC^a^ (No/Yes)	5.803 (1.386–24.286)	0.016	4.653 (0.991–21.844)	0.051
CMV (No/Yes)	1.240 (0.153–10.080)	0.840		
PCTc				
PCT negative	1^b^		1^b^	
PCTc ≥80% or PCT became negative	1.125 (0.117–10.811)	0.919	1.298 (0.112–15.028)	0.835
PCTc <80%	5.239 (1.172–23.421)	0.030	5.697 (1.122–28.931)	0.036

a MTC included III–IV aGVHD, veno-occlusive disease, thrombotic microangiopathy and neurological complications.

b PCT negative group was defined as the control group.

### Prognostic Nomogram for 100-Day Survival

**Figure 4:**
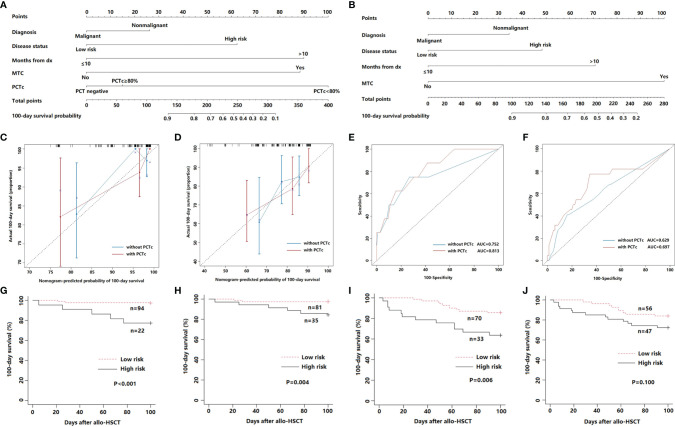
**(A)** Nomogram with PCTc for predicting the 100-day survival of febrile patients in the neutropenic period after allo-HSCT. **(B)** Nomogram without PCTc for predicting the 100-day survival of febrile patients in the neutropenic period after allo-HSCT. **(C, D)** Calibration curve of nomogram with or without PCTc for predicting patient survival at 100 days in the primary **(C)** and validation cohort **(D)**. **(E, F)** ROC curve of nomogram with or without PCTc for the predicted 100-day survival based on the nomogram in the primary **(E)** and validation cohort **(F)**. **(G, H)** 100-day survival according to the nomogram score of nomogram with PCTc in the primary **(G)** and validation cohort **(H)**. **(I, J)** 100-day survival according to the nomogram score without PCTc in the primary **(I)** and validation cohort **(J)**.

The positive factors in the univariate analysis were integrated into a prognostic nomogram (**Figure 4A**). The value of an individual patient is located on each variable axis, and a line is drawn upward to determine the number of points received for each variable value. The sum of these numbers is located on the total point axis, and a line is drawn downward to the survival axes to determine the likelihood of 100-day survival. The calibration plots showed optimal agreement between actual survival and predicted survival in both the primary (**Figure 4C**) and validation sets (**Figure 4D**). The C-indices of the nomogram were 0.808 and 0.691 for the primary and validation cohorts, respectively. The predicted model for 100-day survival probability had an AUC of 0.813 in the primary set (**Figure 4E**), while discrimination was also good in the validation set, with an AUC of 0.697 (**Figure 4F**).

According to the ROC curve of the primary cohort, a nomogram score of 130 was the cutoff value for prognostic stratification. Then, patients with scores below 130 (n = 94) were placed into the low-risk group, while patients with scores above 130 (n = 22) were placed in the high-risk group. The survival curve showed that the 100-day survival of the low-risk group was significantly better than that of the high-risk group (P <0.001) (**Figure 4G**). Furthermore, the same result was observed in the validation cohort (P = 0.004) (**Figure 4H**).

We also developed a nomogram without PCTc (**Figure 4B**) and compared the prediction efficiency of the models with and without PCTc. The C-indices (0.746 in primary and 0.621 in validation cohorts) and AUCs (0.752 in primary and 0.629 in validation cohorts) of the nomogram without PCTc in primary and validation cohorts were all lower than those of the one with PCTc (**Figures 4C–F**). According to the prognostic stratification of the nomogram without PCTc, the 100-day survival of the low-risk group was significantly better than that of the high-risk group (P = 0.006, **Figure 4I**). However, there is no significant difference between low- and high-risk groups in the validation set (P = 0.100, **Figure 4J**). It suggested that adding PCTc into the nomogram could improve the power of the prediction model.

## Discussion

Procalcitonin has been confirmed to have clinical value in patients with neutropenia or undergoing HSCT (1–4, 23–26). Koya et al. reported that the recommended diagnostic cutoff level for bacterial or fungal infection was 0.5 ng/ml in HSCT, with optimal sensitivity, and specificity (3). Sauer et al. showed that PCT, with the threshold set at 1.0 ng/ml, could differentiate patients with sepsis from those without sepsis (26). Our data also indicated a good correlation between PCT and the onset of DI or BSI in the post-HSCT neutropenic period. A value of 0.5 ng/ml is recommended as the positive threshold of DI, and PCT levels above 1.0 ng/ml are highly suggestive of systemic infection.

The diagnostic role of PCT in fungal infection during the post-HSCT neutropenic period is not well established. Roques et al. showed that PCT remained low in neutropenic leukemia patients with invasive pulmonary aspergillosis or mucormycosis, while C-reactive protein and fibrinogen were significantly elevated (27). Ortega et al. reported that patients having fungal infection in neutropenic febrile episodes after HSCT had low PCT levels on the first day of infection, whereas PCT levels above 3 ng/ml had a diagnostic value of invasive aspergillosis when fever persisted for more than 5 days (2). In this study, PCT levels were significantly higher in bacterial infections than in noninfectious events, but there was no significant difference between fungal infections and noninfectious events. However, significantly elevated PCT levels were observed in two patients with fungemia (*Candida tropicalis*: 33.68 ng/ml; *Candida albicans*: 1.15 ng/ml). Similar to previous reports (25, 28), our study also showed that the positive rate of PCT in gram-positive BSIs was significantly lower than in gram-negative BSIs. This difference may be due to the membrane composition of gram-negative and gram-positive bacteria (25).

It has been reported that PCT, as a secondary inflammatory cytokine, might amplify the septic response and exacerbate mortality in an experimental sepsis model (29, 30). The maximum PCT level during a whole course of HSCT was reported as an independent prognostic factor in febrile patients undergoing HSCT (3). Unless daily or more frequent PCT measurements were performed, we could not exactly determine the maximum level of PCT. However, multiple measurements increase the hospitalization expense and risk of BSI. Findings from several studies have shown that PCTc levels below 80% from the baseline to days 3–5 were associated with high early mortality in sepsis patients (8, 10). The relationship between PCTc and the prognosis of patients undergoing allo-HSCT remains unclear.

In our study, there was no significant difference in overall survival according to PCTc. However, a significant difference was observed in post-HSCT 100-day survival. The multivariate analysis revealed that the inability to decrease PCT by more than 80% within 5–7 days was an independent factor with a negative effect on 100-day survival. An increase in PCT or PCTc <80% within the first 5–7 days of PCT positivity indicates that patients may be at risk of uncontrollable infection, which is a major cause of early mortality after allo-HSCT. Conversely, patients with PCTc ≥80% within this timeframe would achieve a favorable 100-day post-HSCT survival, similar to patients who were PCT negative.

We also compared the aGVHD-free survival and the cumulative incidence of III–IV aGVHD within 100 days post-HSCT according to PCTc. Although the difference did not reach statistical significance, the PCT <80% group appears to have a higher risk of III–IV aGVHD than the PCTc ≥80% group. Wagner et al. demonstrated that PCT can impair the function and viability of endothelial cells, resulting in capillary leakage and therapy-refractory hypotension during sepsis (31). The development and severity of aGVHD has been confirmed to be associated with endothelial dysfunction and damage (32). Moreover, severe aGVHD is often resistant to standard treatments and requires additional intensive immunosuppressive treatment, which increases the risk of recurrence of fatal infection.

Previous researches demonstrated that PCT monitoring could evaluate the effectiveness of antibacterial therapies. Physicians could tailor antibiotic strategies in critically ill patients according to PCTc, thereby reducing the duration of antibiotic exposure (5–7). Up to now, there are no data regarding the efficacy and safety of procalcitonin-guided antibiotic treatment in febrile neutropenic patients after allo-HSCT. We retrospectively analyzed the antibiotic use of the PCT-positive group in our study. The patients in the PCTc <80% group showed a higher risk of failure of early de-escalation (within 7 days from the escalated antibiotic treatment) of antibiotics than in PCTc ≥80% group (**Table S2**). In other words, sustained PCT elevation is associated with under- or inadequately-treated infections, and patients should be treated by longer or more intensive antibiotic treatments. Moreover, the patients in the PCTc <80% group experienced longer escalated antibiotic treatment than those in the PCTc ≥80% group.

Currently, nomograms have been developed to predict survival in patients with hematological disease or undergoing HSCT (33–35). In this study, we developed a nomogram to estimate post-HSCT 100-day survival. Internal validation with iterative bootstrapping was performed and showed stable performance. One hundred and three patients from April 2015 to March 2016 were enrolled as a validation cohort. The calibration curve and ROC curve in the validation cohort also showed good predictive accuracy of the proposed nomogram. Furthermore, compared with the nomogram without PCTc, the model including PCTc showed stronger prediction power.

As far as we are aware, this is the first attempt to add PCTc as a prognostic factor into a predictive model, and PCTc showed a strong effect on 100-day survival in febrile allo-HSCT patients. Survival prediction may be improved by incorporating this prognostic factor into future predictive models. Our study has several limitations. First, the data for the primary and validation cohorts were obtained from a single institution and included a relatively small sample. Second, due to the retrospective-nature of the study, we could not identify the maximum PCT value during febrile episodes and compare the prognostic value in febrile neutropenic patients between PCTc and the maximum PCT value. Third, no patients died of underlying disease relapse during the post-HSCT 100 days. Thus, this prognostic model does not account for this parameter having a negative impact on survival and may therefore overestimate survival.

In conclusion, our study demonstrated the diagnostic role of PCT in DI and BSI and the predictive value of PCTc in febrile neutropenic patients as an indicator of mortality within the first 100 days following allo-HSCT. Combining PCTc with clinical parameters could improve the prognostic evaluation of febrile patients in the post-HSCT neutropenic period. Our nomogram may help physicians identify high-risk patients and select appropriate treatment strategies.

## Data Availability Statement

The original contributions presented in the study are included in the article/**Supplementary Material**. Further inquiries can be directed to the corresponding authors.

## Ethics Statement

The studies involving human participants were reviewed and approved by the institutional research ethics committee of the First Affiliated Hospital of Soochow University. Written informed consent to participate in this study was provided by the participants’ legal guardian/next of kin. Written informed consent was obtained from the individual(s), and minor(s)’ legal guardian/next of kin, for the publication of any potentially identifiable images or data included in this article.

## Author Contributions

DW and YX contributed to the conception of the study and manuscript revision. MS, DS, TS, and WX contributed to collecting and performing the data analysis and preparing the manuscript. HQ, SC, YH, XT, MM, and AS contributed to data collection and manuscript revision. All authors listed have made a substantial, direct, and intellectual contribution to the work and approved it for publication.

## Funding

This work was supported by grants from the National Natural Science Foundation of China (81730003, 81870120, 81900151, and 82070187), the Natural Science Foundation of Jiangsu Province (BK20171205 and BK20190176), the Social Development Project of Jiangsu Province (BE2019655), the Jiangsu Province Key R&D Program (BE2019798), the Priority Academic Program Development of Jiangsu Higher Education Institutions (PAPD), the Suzhou Medical Science Innovation Project (SKY2021104), and the National Key Research and Development Program (2019YFC0840604).

## Conflict of Interest

The authors declare that the research was conducted in the absence of any commercial or financial relationships that could be construed as a potential conflict of interest.

## Publisher’s Note

All claims expressed in this article are solely those of the authors and do not necessarily represent those of their affiliated organizations, or those of the publisher, the editors and the reviewers. Any product that may be evaluated in this article, or claim that may be made by its manufacturer, is not guaranteed or endorsed by the publisher.
