# Supplementary material for: The Clinical Value of Procalcitonin in the Neutropenic Period After Allogeneic Hematopoietic Stem Cell Transplantation
Source: Front Immunol. 2022 Apr 25;13:843067. doi: 10.3389/fimmu.2022.843067 (PMC9082027; doi:10.3389/fimmu.2022.843067)
Supplement: Supplementary file 4 [file Table_1.docx]

**Supplemental material**

1. **Conditioning regimen for HSCT and graft-versus-host disease prophylaxis**

For nonmalignant hematological disease, nine patients received Bu/Cy+ATG (IV busulfan (Bu) 3.2 mg/kg/d, days -7 to -6; IV cyclophosphamide (Cy) 50 mg/kg/d, days -5 to -2; and IV rabbit antithymocyte globulin (r-ATG) 2.5 mg/kg/d, days -5 to -2) as a conditioning regimen. Two patients received the FC+ATG regimen (IV fludarabine (Flu) 30 mg/m^2^/d, days -5 to -2; IV Cy 45 mg/kg/d, days -3 to -2; and IV r-ATG 2.5 mg/kg/d, days -5 to -2). For malignant disease, 64 patients received the modified Bu/Cy regimen (IV Ara-c 2 g/m^2^/d, days -9 to -8; IV Bu 3.2 mg/kg/d, days -7 to -5; IV Cy 1.8 g/m^2^/d, days -4 to -3; and oral semustine 250 mg/m^2^/d, day -10). Thirty-two malignant patients received the Bu/Cy regimen (IV Bu 3.2 mg/kg/d, days -7 to -4, and IV Cy 60 mg/kg/d days -3 to -2). Nine malignant patients received the TBI/Cy regimen (total body irradiation (TBI) 4 Gy/d, days -3 to -1, and IV Cy 60 mg/kg/d, days -6 to -5). r-ATG (IV 2.5 mg/kg/d, days -5 to -2) was included in the conditioning regimen of 84 malignant hematological patients.

Graft-versus-host disease (GVHD) prophylaxis regimens consisting of cyclosporine (CsA, 2.5–3 mg/kg/d, days -1 to +30, then tapering) and short-range methotrexate (MTX) (15 mg/m^2^/d, day +1 and 10 mg/m2/d, days +3,+6 and +11) for HLA-matched donors, while CsA (2.5–3 mg/kg/d, days -9 to +30, then tapering), MTX (15 mg/m^2^/d, day +1 and 10 mg/m^2^/d, days +3,+6 and +11), mycophenolate mofetil (MMF, 1.0 twice a day, days -10 to +30, then tapering) were used for HLA-mismatched donors.

**Table S1.** **Engraftment and** **transplant-related complications in the primary cohort**

|  | **PCTc <80%** | **PCTc ≥80%** | **PCT negative** | **p-value** |
| --- | --- | --- | --- | --- |
|  | **n=21** | **n=22** | **n=73** |  |
| **Engraftment** |  |  |  |  |
| Time to ANC ≥ 0.5*10^9^/L, days | 12 (9-18)**^a^** | 12 (10-19) | 12 (9-22) | 0.232 |
| Time to platelet ≥ 20*10^9^/L, days | 13 (10-50)**^a^** | 18 (9-46) | 14 (8-56) | 0.205 |
| **Transplant-related complications, n (%)** |  |  |  |  |
| BSI | 10 (47.6) | 8 (36.4) | 8 (11.0) | <0.001 |
| aGVHD |  |  |  | 0.109 |
| Grade I-II | 4 (19.0) | 7 (31.8) | 29 (39.7) |  |
| Grade III-IV | 6 (28.6) | 2 (9.1) | 10 (13.7) |  |
| cGVHD |  |  |  | 0.698 |
| Limited | 7 (33.3) | 8 (36.4) | 30 (41.1) |  |
| Extensive | 3 (14.3) | 1 (4.5) | 4 (5.5) |  |
| Veno-occlusive disease | 1 (4.8) | 0 (0) | 0 (0) | 0.181 |
| Hemorrhagic cystitis | 3 (14.3) | 4 (18.2) | 13 (17.8) | >0.999 |
| Thrombotic microangiopathy | 0 (0) | 0 (0) | 1 (1.4) | >0.999 |
| Engraftment syndrome | 0 (0) | 1 (9.1) | 1 (1.4) | >0.999 |
| Neurological complications**^b^** | 2 (9.5) | 1 (9.1) | 6 (8.2) | 0.892 |
| CMV | 0 (0) | 3 (13.6) | 9 (12.3) | 0.269 |
| EBV | 2 (9.5) | 4 (18.2) | 8 (11.0) | 0.706 |

**^a^**Three patients died without neutrophil and platelet implantation.

**^b^**Neurological complications include epilepsy, posterior reversible encephalopathy syndrome and immune-mediated neuropathy.

Abbreviations: ANC, absolute neutrophil count; BSI, bloodstream infection; aGVHD, acute graft-versus-host disease; cGVHD, chronic graft-versus-host disease; CMV, cytomegalovirus; EBV, Epstein-Barr virus.

**Table S2. The adjustment of empirical antibiotic treatment in PCT-positive groups of primary cohort**

|  | | **PCTc ≥80%** |  | **PCTc <80%** |
| --- | --- | --- | --- | --- |
|  | | **n=22** |  | **n=21** |
| **First escalated antibiotic treatment** | | 21/22 |  | 19/22 |
| Duration of escalated antibiotic treatment, days | 10 (6-20) | |  | 12 (5-22) |
| Early de-escalation of antibiotic treatment, n | | 5/21 |  | 1/19 |
| Failure of early de-escalation, n | | 1/5 |  | 1/1 |
| Late de-escalation of antibiotic treatment, n | | 16/21 |  | 18/19 |
| Failure of late de-escalation, n | | 0 |  | 1/18 |
| Time between de-escalation and re-escalation of antibiotic treatment, days | | 6 |  | 4 (3-5) |
| **Second escalated antibiotic treatment** | | 1/22 |  | 2/21 |
| Duration of escalated antibiotic treatment, days | | 10 |  | 17 (6-28) |
| Early de-escalation of antibiotic treatment, n | | 0 |  | 1/2 |
| Failure of early de-escalation, n | | 0 |  | 0 |
| Late de-escalation of antibiotic treatment, n | | 1/1 |  | 1/2 |
| Failure of late de-escalation, n | | 0 |  | 0 |
| Time between de-escalation and re-escalation of antibiotic treatment, days | | 6 |  | 4 (3-5) |
| Dead during escalated antibiotic treatment,n | | 1/23 |  | 2/23 |
