# Supplementary material for: The Clinical Value of Procalcitonin in the Neutropenic Period After Allogeneic Hematopoietic Stem Cell Transplantation
Source: Front Immunol. 2022 Apr 25;13:843067. doi: 10.3389/fimmu.2022.843067 (PMC9082027; doi:10.3389/fimmu.2022.843067)
Supplement: Supplementary file 5 [file Table_2.docx]

**1. R script for nomogram without PCTc**

library(rms)

library(foreign)

library(survival)

dev<-read.csv("dev.csv")

head(dev)

str(dev)

dev$Diagnosis<-factor(dev$Diagnosis,labels=c('Malignant','Nomalignant'))

dev$Disease.status<-factor(dev$Disease.status,labels=c('Low risk','High risk'))

dev$interval<-factor(dev$interval,labels=c('≤10','>10')) # months from diagnosis

dev$MTC<-factor(dev$MTC,labels=c('No','Yes'))

str(dev)

ddist <- datadist(dev)

options(datadist='ddist')

units(dev$follow) <- "Day"

fcox <- cph(Surv(follow,death) ~ Diagnosis + Disease.status + interval + MTC,surv=T,x=T, y=T,time.inc = 100, data=dev)

fcox

surv<-Survival(fcox)

surv1<-function(x)surv(1*100,lp=x)

plot(nomogram(fcox,fun = list(surv1),lp=F,funlabel = c("100-day survival probability"),maxscale = 100,fun.at = c("0.9","0.8","0.7", "0.6","0.5","0.4","0.3","0.2")),xfrac = .45)

**2. R script for nomogram with PCTc**

library(rms)

library(foreign)

library(survival)

dev<-read.csv("dev.csv")

head(dev)

str(dev)

dev$Diagnosis<-factor(dev$Diagnosis,labels=c('Malignant','Nomalignant'))

dev$Disease.status<-factor(dev$Disease.status,labels=c('Low risk','High risk'))

dev$interval<-factor(dev$interval,labels=c('≤10','>10')) # months from diagnosis

dev$MTC<-factor(dev$MTC,labels=c('No','Yes'))

dev$PCTc<-factor(dev$PCTc,labels=c('Negative','PCTc≥80%','PCTc<80%'))

str(dev)

ddist <- datadist(dev)

options(datadist='ddist')

units(dev$follow) <- "Day"

fcox1 <- cph(Surv(follow,death) ~ Diagnosis + Disease.status + interval + MTC + PCTc,surv=T,x=T, y=T,time.inc = 100, data=dev)

fcox1

surv<-Survival(fcox1)

surv2<-function(x)surv(1*100,lp=x)

plot(nomogram(fcox1,fun = list(surv2),lp=F,funlabel = c("100-day survival probability"),maxscale = 100,fun.at = c("0.9","0.8","0.7", "0.6","0.5","0.4","0.3","0.2","0.1")),xfrac = .45)

**3. R script for calibration curves of nomogram with or without PCTc for predicting patient survival at 100 days in the primary cohort**

dev<-read.csv("dev.csv")

head(dev)

str(dev)

dev$Diagnosis<-factor(dev$Diagnosis,labels=c('Malignant','Nomalignant'))

dev$Disease.status<-factor(dev$Disease.status,labels=c('Low risk','High risk'))

dev$interval<-factor(dev$interval,labels=c('≤10','>10')) #months from diagnosis

dev$MTC<-factor(dev$MTC,labels=c('No','Yes'))

dev$PCTc<-factor(dev$PCTc,labels=c('Negative','PCTc≥80%','PCTc<80%'))

str(dev)

ddist <- datadist(dev)

options(datadist='ddist')

units(dev$follow) <- "Day"

fcox <- cph(Surv(follow,death) ~ Diagnosis + Disease.status + interval + MTC,surv=T,x=T, y=T,time.inc = 100, data=dev)

f <- cph(Surv(follow,death) ~ predict(fcox, newdata=dev), x=T, y=T, surv=T, data=dev)

validate(f, method="boot", B=500, dxy=T)

rcorrcens(Surv(follow,death) ~ predict(fcox, newdata=dev), data = dev)

cal <- calibrate(fcox, cmethod="KM", method="boot", u=100, m=30, B=500)

plot(cal, lwd=2, lty=1,errbar.col=c(rgb(0,118,192,maxColorValue=255)), xlim=c(0.7,1), ylim=c(0.7,1),col=c(rgb(192,98,83,maxColorValue=255)))

abline(0,1,lty=3,lwd=2,col=c(rgb(0,0,0,maxColorValue=255)))

fcox1 <- cph(Surv(follow,death) ~ Diagnosis + Disease.status + interval + MTC+ PCTc, surv=T,x=T, y=T,time.inc = 100, data=dev)

f1 <- cph(Surv(follow,death) ~ predict(fcox1, newdata=dev), x=T, y=T, surv=T, data=dev)

validate(f1, method="boot", B=500, dxy=T)

rcorrcens(Surv(follow,death) ~ predict(fcox1, newdata=dev), data = dev)

cal1 <- calibrate(fcox1, cmethod="KM", method="boot", u=100, m=30, B=500)

plot(cal, lwd=2, lty=1,errbar.col=c(rgb(0,118,192,maxColorValue=255)), xlim=c(0.7,1), ylim=c(0.7,1),col=c(rgb(0,118,192,maxColorValue=255)))

abline(0,1,lty=3,lwd=2,col=c(rgb(0,0,0,maxColorValue=255)))

plot(cal1, add=T, lwd=2, lty=1,errbar.col=c(rgb(176,23,31,maxColorValue=255)), xlim=c(0.7,1), ylim=c(0.7,1),col=c(rgb(176,23,31,maxColorValue=255)))

**4. R script for calibration curves of nomogram with or without PCTc for predicting patient survival at 100 days in the validation cohort**

vad<-read.csv("vad.csv")

head(vad)

str(vad)

vad$Diagnosis<-factor(vad$Diagnosis,labels=c('Malignant','Nomalignant'))

vad$Disease.status<-factor(vad$Disease.status,labels=c('Low risk','High risk'))

vad$interval<-factor(vad$interval,labels=c('≤10','>10'))

vad$MTC<-factor(vad$MTC,labels=c('No','Yes'))

vad$PCTc<-factor(vad$PCTc,labels=c('Negative','PCTc≥80%','PCTc<80%'))

str(vad)

fvad <-cph(Surv(follow, death) ~ predict(fcox,newdata=vad),x=T, y=T,surv=T, data=vad)

validate(fvad, method="boot", B=500, dxy=T)

rcorrcens(Surv(follow, death) ~ predict(fcox, newdata=vad), data = vad)

fvad1 <-cph(Surv(follow, death) ~ predict(fcox1,newdata=vad),x=T, y=T,surv=T, data=vad)

validate(fvad1, method="boot", B=500, dxy=T)

rcorrcens(Surv(follow, death) ~ predict(fcox1, newdata=vad), data = vad)

ddist <- datadist(vad)

options(datadist='ddist')

units(vad$follow) <- "Day"

fvad <- cph(Surv(follow, death) ~predict(fcox, newdata=vad), x=T, y=T, surv=T, data=vad, time.inc=100)

cfvad <- calibrate(fvad, cmethod="KM", method="boot", u=100, m=30, B=500)

fvad1 <- cph(Surv(follow, death) ~predict(fcox1, newdata=vad), x=T, y=T, surv=T, data=vad, time.inc=100)

cfvad1 <- calibrate(fvad1, cmethod="KM", method="boot", u=100, m=30, B=500)

plot(cfvad, lwd=2, lty=1,errbar.col=c(rgb(0,118,192,maxColorValue=255)), xlim=c(0.4,1), ylim=c(0.4,1),col=c(rgb(0,118,192,maxColorValue=255)))

abline(0,1,lty=3,lwd=2,col=c(rgb(0,0,0,maxColorValue=255)))

plot(cfvad1, add=T, lwd=2, lty=1,errbar.col=c(rgb(176,23,31,maxColorValue=255)), xlim=c(0.4,1), ylim=c(0.4,1),col=c(rgb(176,23,31,maxColorValue=255)))

**5. R script for ROC curves of nomogram with or without PCTc in the primary cohort**

library(rms)

library(foreign)

library(survival)

dev<-read.csv("dev.csv")

View(dev)

dev$Diagnosis<-factor(dev$Diagnosis,labels=c('Malignant','Nomalignant'))

dev$Disease.status<-factor(dev$Disease.status,labels=c('Low risk','High risk'))

dev$interval<-factor(dev$interval,labels=c('≤10','>10'))

dev$MTC<-factor(dev$MTC,labels=c('No','Yes'))

dev$PCTc<-factor(dev$PCTc,labels=c('Negative','PCTc≥80%','PCTc<80%'))

str(dev)

library(survivalROC)

nobs<- NROW(dev)

cutoff1<- 100

Srv=Surv(dev$follow,dev$death)

coxmod=coxph(Srv ~ Diagnosis + Disease.status + interval + MTC ,data=dev)

summary(coxmod)

Diagnosisn<- as.numeric(dev$Diagnosis)

dev$Diagnosispoint<- ifelse(Diagnosisn==1,0,0.6286)

Disease.statusn<- as.numeric(dev$Disease.status)

dev$Disease.statuspoint<- ifelse(Disease.statusn==1,0,0.8847)

MTCn<- as.numeric(dev$MTC)

dev$MTCpoint<- ifelse(MTCn==1,0,1.8325)

intervaln<- as.numeric(dev$interval)

dev$intervalpoint<- ifelse(intervaln==1,0,1.2960)

dev$PI <- dev$points <- rowSums(dev[,c("Diagnosispoint","Disease.statuspoint","intervalpoint","MTCpoint")])

summary(dev$PI)

data<-dev[which(dev$death!="NA"),]

SROC= survivalROC(Stime = data$follow, status = data$death, marker = data$PI, predict.time =cutoff1, method= "KM" )

cut.op= SROC$cut.values[which.max(SROC$TP-SROC$FP)]

cut.op

plot(SROC$FP,SROC$TP, type="l", xlim=c(0,1), ylim=c(0,1), xlab = paste( "FP","\n", "AUC = ",round(SROC$AUC,3)), ylab = "TP", col="red")

abline(0,1)

Srv1=Surv(dev$follow,dev$death)

coxmod1=coxph(Srv1 ~ Diagnosis + Disease.status + interval + MTC + PCTc ,data=dev)

summary(coxmod1)

Diagnosisn<- as.numeric(dev$Diagnosis)

dev$Diagnosispoint1<- ifelse(Diagnosisn==1,0,0.4540)

Disease.statusn<- as.numeric(dev$Disease.status)

dev$Disease.statuspoint1<- ifelse(Disease.statusn==1,0,1.0844)

MTCn<- as.numeric(dev$MTC)

dev$MTCpoint1<- ifelse(MTCn==1,0,1.5375)

intervaln<- as.numeric(dev$interval)

dev$intervalpoint1<- ifelse(intervaln==1,0,1.5651)

PCTcn<- as.numeric(dev$PCTc)

dev$PCTcpoint1<-ifelse(PCTcn==1,0,ifelse(PCTcn==2,0.2610,1.7400))

dev$PI1 <- dev$points1 <- rowSums(dev[,c("Diagnosispoint1","Disease.statuspoint1","intervalpoint1","MTCpoint1","PCTcpoint1")])

summary(dev$PI1)

data<-dev[which(dev$death!="NA"),]

SROC1= survivalROC(Stime = data$follow, status = data$death, marker = data$PI1, predict.time =cutoff1, method= "KM" )

cut.op= SROC1$cut.values[which.max(SROC1$TP-SROC1$FP)]

cut.op

plot(SROC1$FP,SROC1$TP, type="l", xlim=c(0,1), ylim=c(0,1), xlab = paste( "FP","\n", "AUC = ",round(SROC1$AUC,3)), ylab = "TP", col="red")

abline(0,1)

require(ggsci)

library("scales")

pal_nejm("default")(8)

show_col(pal_nejm("default")(8))

plot(SROC$FP, SROC$TP, ## x=FP,y=TP

type="l",col="#0072B5FF",

xlim=c(0,1), ylim=c(0,1),

xlab=("1 - Specificity"),

ylab="Sensitivity")

abline(0,1,col="black",lty=2,)

lines(SROC1$FP, SROC1$TP, type="l",col="#BC3C29FF",xlim=c(0,1), ylim=c(0,1))

legend(0.45,0.3,c(paste("AUC",round(SROC$AUC,100)),paste("AUC of 1",round(SROC1$AUC,100))),x.intersp = 1, y.intersp = 0.8, lty = 1,lwd = 2, col = c("#0072B5FF","#BC3C29FF"),bty = "n",seg.len = 1,cex = 0.8)

**6. R script for ROC curves of nomogram with or without PCTc in the validation cohort**

library(rms)

library(foreign)

library(survival)

vad<-read.csv("vad.csv")

View(vad)

vad$Diagnosis<-factor(vad$Diagnosis,labels=c('Malignant','Nomalignant'))

vad$Disease.status<-factor(vad$Disease.status,labels=c('Low risk','High risk'))

vad$interval<-factor(vad$interval,labels=c('≤10','>10'))

vad$MTC<-factor(vad$MTC,labels=c('No','Yes'))

vad$PCTc<-factor(vad$PCTc,labels=c('Negative','PCTc≥80%','PCTc<80%'))

str(vad)

library(survivalROC)

nobs<- NROW(vad)

cutoff1<- 100

Diagnosisn<- as.numeric(vad$Diagnosis)

vad$Diagnosispoint<- ifelse(Diagnosisn==1,0,0.6286)

Disease.statusn<- as.numeric(vad$Disease.status)

vad$Disease.statuspoint<- ifelse(Disease.statusn==1,0,0.8847)

MTCn<- as.numeric(vad$MTC)

vad$MTCpoint<- ifelse(MTCn==1,0,1.8325)

intervaln<- as.numeric(vad$interval)

vad$intervalpoint<- ifelse(intervaln==1,0,1.2960)

vad$PI <- vad$points <- rowSums(vad[,c("Diagnosispoint","Disease.statuspoint","intervalpoint","MTCpoint")])

summary(vad$PI)

data<-vad[which(vad$death!="NA"),]

SROC= survivalROC(Stime = data$follow, status = data$death, marker = data$PI, predict.time =cutoff1, method= "KM" )

plot(SROC$FP,SROC$TP, type="l", xlim=c(0,1), ylim=c(0,1), xlab = paste( "FP","\n", "AUC = ",round(SROC$AUC,3)), ylab = "TP", col="red")

abline(0,1)

Diagnosisn<- as.numeric(vad$Diagnosis)

vad$Diagnosispoint1<- ifelse(Diagnosisn==1,0,0.4540)

Disease.statusn<- as.numeric(vad$Disease.status)

vad$Disease.statuspoint1<- ifelse(Disease.statusn==1,0,1.0844)

MTCn<- as.numeric(vad$MTC)

vad$MTCpoint1<- ifelse(MTCn==1,0,1.5375)

intervaln<- as.numeric(vad$interval)

vad$intervalpoint1<- ifelse(intervaln==1,0,1.5651)

PCTcn<- as.numeric(vad$PCTc)

vad$PCTcpoint1<-ifelse(PCTcn==1,0,ifelse(PCTcn==2,0.2610,1.7400))

vad$PI1 <- vad$points1 <- rowSums(vad[,c("Diagnosispoint1","Disease.statuspoint1","intervalpoint1","MTCpoint1","PCTcpoint1")])

summary(vad$PI1)

data<-vad[which(vad$death!="NA"),]

SROC1= survivalROC(Stime = data$follow, status = data$death, marker = data$PI1, predict.time =cutoff1, method= "KM" )

plot(SROC1$FP,SROC1$TP, type="l", xlim=c(0,1), ylim=c(0,1), xlab = paste( "FP","\n", "AUC = ",round(SROC1$AUC,3)), ylab = "TP", col="red")

abline(0,1)

require(ggsci)

library("scales")

pal_nejm("default")(8)

show_col(pal_nejm("default")(8))

plot(SROC$FP, SROC$TP, ## x=FP,y=TP

type="l",col="#0072B5FF",

xlim=c(0,1), ylim=c(0,1),

xlab=("1 - Specificity"),

ylab="Sensitivity")

abline(0,1,col="black",lty=2,)

lines(SROC1$FP, SROC1$TP, type="l",col="#BC3C29FF",xlim=c(0,1), ylim=c(0,1))

legend(0.45,0.3,c(paste("AUC",round(SROC$AUC,100)),paste("AUC of 1",round(SROC1$AUC,100))),x.intersp = 1, y.intersp = 0.8, lty = 1,lwd = 2, col = c("#0072B5FF","#BC3C29FF"),bty = "n",seg.len = 1,cex = 0.8)

**7. R script for Kaplan-Meier curves according to the score of nomogram without PCTc in the primary and validation cohorts**

library(rms)

library(foreign)

library(survival)

dev<-read.csv("dev.csv")

View(dev)

dev$Diagnosis<-factor(dev$Diagnosis,labels=c('Malignant','Nomalignant'))

dev$Disease.status<-factor(dev$Disease.status,labels=c('Low risk','High risk'))

dev$interval<-factor(dev$interval,labels=c('≤10','>10'))

dev$MTC<-factor(dev$MTC,labels=c('No','Yes'))

str(dev)

library(survivalROC)

nobs<- NROW(dev)

cutoff1<- 100

Srv=Surv(dev$follow,dev$death)

coxmod=coxph(Srv ~ Diagnosis + Disease.status + interval + MTC ,data=dev)

summary(coxmod)

Diagnosisn<- as.numeric(dev$Diagnosis)

dev$Diagnosispoint<- ifelse(Diagnosisn==1,0,0.6286)

Disease.statusn<- as.numeric(dev$Disease.status)

dev$Disease.statuspoint<- ifelse(Disease.statusn==1,0,0.8847)

intervaln<- as.numeric(dev$interval)

dev$intervalpoint<- ifelse(intervaln==1,0,1.2960)

MTCn<- as.numeric(dev$MTC)

dev$MTCpoint<- ifelse(MTCn==1,0,1.8325)

dev$PI <- dev$points <- rowSums(dev[,c("Diagnosispoint","Disease.statuspoint","intervalpoint","MTCpoint")])

summary(dev$PI)

data<-dev[which(dev$death!="NA"),]

SROC= survivalROC(Stime = data$follow, status = data$death, marker = data$PI, predict.time =cutoff1, method= "KM" )

cut.op= SROC$cut.values[which.max(SROC$TP-SROC$FP)]

cut.op

dev$PIgroup<- ifelse(dev$PI<=1.296,0,1)

fit <- survfit(Surv(follow,death)~ PIgroup,data=dev)

plot(fit,lty=c(2,1),col=c(2,1),mark.time = T)

legend('bottomright',legend=c('low risk','hight risk'),lty=c(2,1),col=c(2,1))

survdiff(Surv(follow,death)~ PIgroup,data=dev, rho = 0)

vad<-read.csv("vad.csv")

View(vad)

vad$Diagnosis<-factor(vad$Diagnosis,labels=c('Malignant','Nomalignant'))

vad$Disease.status<-factor(vad$Disease.status,labels=c('Low risk','High risk'))

vad$interval<-factor(vad$interval,labels=c('≤10','>10'))

vad$MTC<-factor(vad$MTC,labels=c('No','Yes'))

str(vad)

nobs<- NROW(vad)

cutoff1<- 100

Diagnosisn<- as.numeric(vad$Diagnosis)

vad$Diagnosispoint<- ifelse(Diagnosisn==1,0,0.6286)

Disease.statusn<- as.numeric(vad$Disease.status)

vad$Disease.statuspoint<- ifelse(Disease.statusn==1,0,0.8847)

intervaln<- as.numeric(vad$interval)

vad$intervalpoint<- ifelse(intervaln==1,0,1.2960)

MTCn<- as.numeric(vad$MTC)

vad$MTCpoint<- ifelse(MTCn==1,0,1.8325)

vad$PI <- vad$points <- rowSums(vad[,c("Diagnosispoint","Disease.statuspoint","intervalpoint","MTCpoint")])

summary(vad$PI)

vad$PIgroup<- ifelse(vad$PI<=1.296,0,1)

fit <- survfit(Surv(follow,death)~ PIgroup,data=vad)

plot(fit,lty=c(2,1),col=c(2,1),mark.time = T)

legend('bottomright',legend=c('low risk','hight risk'),lty=c(2,1),col=c(2,1))

survdiff(Surv(follow,death)~ PIgroup,data=vad, rho = 0)

**8. R script for Kaplan-Meier curves according to the score of nomogram with PCTc in the primary and validation cohorts**

library(rms)

library(foreign)

library(survival)

dev<-read.csv("dev.csv")

View(dev)

dev$Diagnosis<-factor(dev$Diagnosis,labels=c('Malignant','Nomalignant'))

dev$Disease.status<-factor(dev$Disease.status,labels=c('Low risk','High risk'))

dev$interval<-factor(dev$interval,labels=c('≤10','>10'))

dev$MTC<-factor(dev$MTC,labels=c('No','Yes'))

dev$PCTc<-factor(dev$PCTc,labels=c('Negative','PCTc≥80%','PCTc<80%'))

str(dev)

library(survivalROC)

nobs<- NROW(dev)

cutoff1<- 100

Srv1=Surv(dev$follow,dev$death)

coxmod1=coxph(Srv1 ~ Diagnosis + Disease.status + interval + MTC + PCTc ,data=dev)

summary(coxmod1)

Diagnosisn<- as.numeric(dev$Diagnosis)

dev$Diagnosispoint1<- ifelse(Diagnosisn==1,0,0.4540)

Disease.statusn<- as.numeric(dev$Disease.status)

dev$Disease.statuspoint1<- ifelse(Disease.statusn==1,0,1.0844)

intervaln<- as.numeric(dev$interval)

dev$intervalpoint1<- ifelse(intervaln==1,0,1.5651)

MTCn<- as.numeric(dev$MTC)

dev$MTCpoint1<- ifelse(MTCn==1,0,1.5375)

PCTcn<- as.numeric(dev$PCTc)

dev$PCTcpoint1<-ifelse(PCTcn==1,0,ifelse(PCTcn==2,0.2610,1.7400))

dev$PI1 <- dev$points1 <- rowSums(dev[,c("Diagnosispoint1","Disease.statuspoint1","intervalpoint1","MTCpoint1","PCTcpoint1")])

summary(dev$PI1)

data<-dev[which(dev$death!="NA"),]

SROC1= survivalROC(Stime = data$follow, status = data$death, marker = data$PI1, predict.time =cutoff1, method= "KM" )

cut.op= SROC1$cut.values[which.max(SROC1$TP-SROC1$FP)]

cut.op

dev$PI1group<- ifelse(dev$PI1<=2.2801,0,1)

fit1 <- survfit(Surv(follow,death)~ PI1group,data=dev)

plot(fit1,lty=c(2,1),col=c(2,1),mark.time = T)

legend('bottomright',legend=c('low risk','hight risk'),lty=c(2,1),col=c(2,1))

survdiff(Surv(follow,death)~ PI1group,data=dev, rho = 0)

vad<-read.csv("vad.csv")

View(vad)

vad$Diagnosis<-factor(vad$Diagnosis,labels=c('Malignant','Nomalignant'))

vad$Disease.status<-factor(vad$Disease.status,labels=c('Low risk','High risk'))

vad$interval<-factor(vad$interval,labels=c('≤10','>10'))

vad$MTC<-factor(vad$MTC,labels=c('No','Yes'))

vad$PCTc<-factor(vad$PCTc,labels=c('Negative','PCTc≥80%','PCTc<80%'))

str(vad)

nobs<- NROW(vad)

cutoff1<- 100

Diagnosisn<- as.numeric(vad$Diagnosis)

vad$Diagnosispoint1<- ifelse(Diagnosisn==1,0,0.4540)

Disease.statusn<- as.numeric(vad$Disease.status)

vad$Disease.statuspoint1<- ifelse(Disease.statusn==1,0,1.0844)

intervaln<- as.numeric(vad$interval)

vad$intervalpoint1<- ifelse(intervaln==1,0,1.5651)

MTCn<- as.numeric(vad$MTC)

vad$MTCpoint1<- ifelse(MTCn==1,0,1.5375)

PCTcn<- as.numeric(vad$PCTc)

vad$PCTcpoint1<-ifelse(PCTcn==1,0,ifelse(PCTcn==2,0.2610,1.7400))

vad$PI1 <- vad$points1 <- rowSums(vad[,c("Diagnosispoint1","Disease.statuspoint1","intervalpoint1","MTCpoint1","PCTcpoint1")])

summary(vad$PI1)

vad$PI1group<- ifelse(vad$PI1<=2.2801,0,1)

fit1 <- survfit(Surv(follow,death)~ PI1group,data=vad)

plot(fit1,lty=c(2,1),col=c(2,1),mark.time = T)

legend('bottomright',legend=c('low risk','hight risk'),lty=c(2,1),col=c(2,1))

survdiff(Surv(follow,death)~ PI1group,data=vad, rho = 0)
